# Spatial variation in soil properties and diffuse losses between and within grassland fields with similar short‐term management

**DOI:** 10.1111/ejss.12351

**Published:** 2016-07-15

**Authors:** S. Peukert, B. A. Griffith, P. J. Murray, C. J. A. Macleod, R. E. Brazier

**Affiliations:** ^1^ College of Life and Environmental SciencesUniversity of ExeterAmory Building, Rennes DriveExeter EX4 4RJUK; ^2^ Sustainable Soils and Grassland Systems DepartmentRothamsted ResearchNorth WykeOkehampton EX20 2SBUK; ^3^ Information and Computational Sciences GroupThe James Hutton InstituteCraigiebucklerAberdeen AB15 8QHUK

## Abstract

One of the major challenges for agriculture is to understand the effects of agricultural practices on soil properties and diffuse pollution, to support practical farm‐scale land management. Three conventionally managed grassland fields with similar short‐term management, but different ploughing histories, were studied on a long‐term research platform: the North Wyke Farm Platform. The aims were to (i) quantify the between‐field and within‐field spatial variation in soil properties by geostatistical analysis, (ii) understand the effects of soil condition (in terms of nitrogen, phosphorus and carbon contents) on the quality of discharge water and (iii) establish robust baseline data before the implementation of various grassland management scenarios. Although the fields sampled had experienced the same land use and similar management for at least 6 years, there were differences in their mean soil properties. They showed different patterns of soil spatial variation and different rates of diffuse nutrient losses to water. The oldest permanent pasture field had the largest soil macronutrient concentrations and the greatest diffuse nutrient losses. We show that management histories affect soil properties and diffuse losses. Potential gains in herbage yield or benefits in water quality might be achieved by characterizing every field or by area‐specific management within fields (a form of precision agriculture for grasslands). Permanent pasture per se cannot be considered a mitigation measure for diffuse pollution. The between‐ and within‐field soil spatial variation emphasizes the importance of baseline characterization and will enable the reliable identification of any effects of future management change on the Farm Platform.

**Highlights:**

- Quantification of soil and water quality in grassland fields with contrasting management histories.
- Considerable spatial variation in soil properties and diffuse losses between and within fields.
- Contrasting management histories within and between fields strongly affected soil and water quality.
- Careful pasture management needed: the oldest pasture transferred the most nutrients from soil to water.

## Introduction

Agricultural practices inevitably affect soil physical properties, plant nutrient concentrations and the potential to optimize or maximize crop production. Soil properties that are changed, subsequently affect ecosystem services such as nutrient sources, and their mobilization and delivery to surface waters. Many mitigation measures for nutrient pollution have been implemented in the past two decades, most of which aim to improve soil quality and minimize the mobilization of nutrients from agricultural land. Conversion from arable to permanent grassland, soil aeration and precision agriculture are examples of measures that can mitigate nutrient pollution. The effectiveness of these measures appears slow to emerge (Johnson *et al*., 2011; van Grinsven *et al.*, 2012).

Effectiveness of mitigation measures can be difficult to detect because the legacy of intensive agriculture takes time to affect soil and water quality (Horrocks *et al.*, 2014). Therefore, soil status and the quality of surface waters today reflect, to some extent, past management rather than current management (Burt *et al.*, 2011). Disentangling the relation between short‐ and long‐term management is crucial to understanding the effect of management on soil health and water quality.

Improvements in soil and water quality are often evaluated separately, even though both operate along a continuum from soil to water. An alternative approach to study the effects of management could include the entire source‐mobilization‐delivery continuum to identify the continuum components that have improved and those that need further improvement (Wall *et al.*, 2011). For example, Johnson *et al.* (2011) showed that plant nutrient surpluses in grasslands had been reduced, but there was no overall reduction in nutrient concentrations in surface water. This suggested that potential nutrient sources might have reduced, but their mobilization rates needed further mitigation efforts.

Detecting an improvement in soil and water quality by management is often limited by poor understanding of the conventional pre‐mitigation management, to which post‐mitigation data can be compared. Before any new management practice can be quantified, a baseline understanding or characterization of the *status quo* of the conventional management of both soil properties and resulting water quality is essential (Breuer *et al.*, 2006). Some water quality studies use paired catchment studies that involve baseline characterization (Schilling *et al.*, 2013). Soil studies, however, often compare soil properties between sites with different land uses without baseline characterization and rely on the fundamental assumption that the sites were similar in terms of soil properties prior to land‐use change (Breuer *et al.*, 2006). We consider here that incorrect inferences might be made about management effects without analysing those effects in relation to baseline conditions.

It is not just the average soil condition in a field that might change as a result of management, but also the spatial distribution of soil properties. Spatial variation exists in agricultural fields in spite of uniform fertilizer applications, uniform vegetation cover and uniform soil physical management within fields (McCormick *et al.*, 2009; Peukert *et al.*, 2012). This spatial variation needs to be quantified both *between* and *within* agricultural fields so that any change in either the overall mean or in the spatial distribution of soil properties can be attributed to changes in management (Glendell *et al.*, 2014). Consequently, it is necessary to adopt an appropriate sampling strategy to achieve this.

Conventionally managed grassland fields with similar short‐term management, but differences in long‐term management history, were studied to address the following objectives.

1. To characterize within‐ and between‐field spatial variation of soil physical and chemical properties in an intensively managed grassland.
2. To characterize between‐field differences in hydrology and water quality in terms of sediment and the macronutrients N, C and P in relation to between‐field differences in soil properties and other site characteristics (topography and management),
3. To establish a suitable baseline characterization between future control and treatment sites in terms of soil and water properties.

## Methods

**Figure 1:**
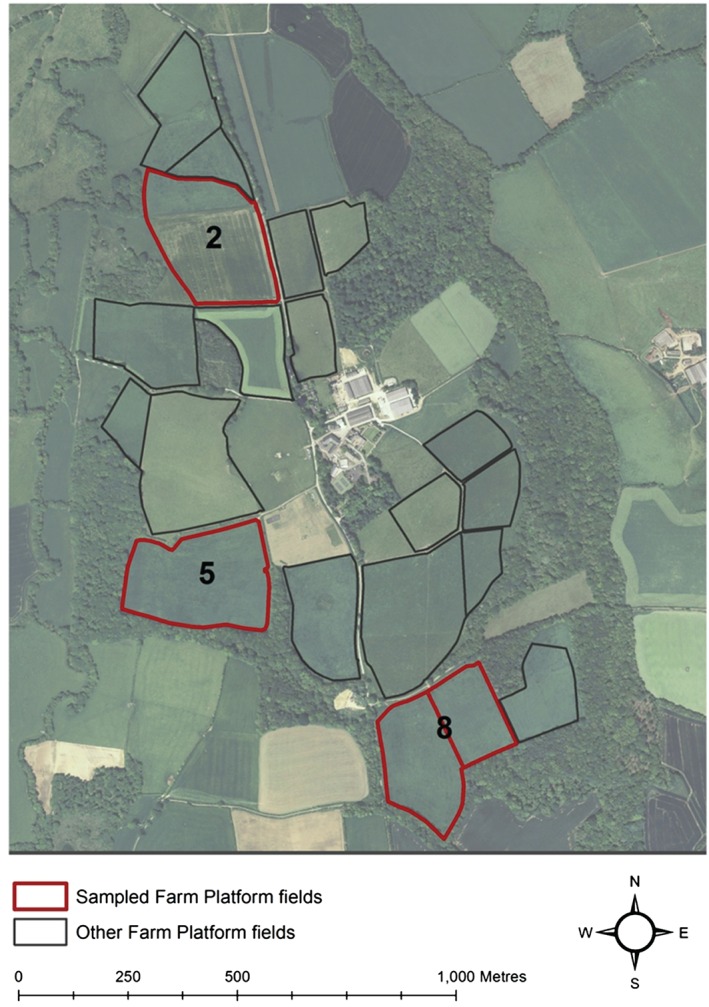
Location of the North Wyke Farm Platform and the three fields sampled: field 2, field 5 and field 8.

Soil and water were sampled on the Rothamsted Research ‘North Wyke Farm Platform’ in southwest England (Figure 1). The soil was sampled in June 2012 and water was sampled between April 2012 and 2013. The Farm Platform comprises 15 fields subdivided into three equal‐sized farmlets that were under similar management during the sampling period, but which will be subjected to different management strategies in the future. Each Farm Platform field is hydrologically isolated so that water leaving the field by subsurface and surface flow is channelled by French drains into flumes, where both water quantity and quality are monitored.

**Figure 2:**
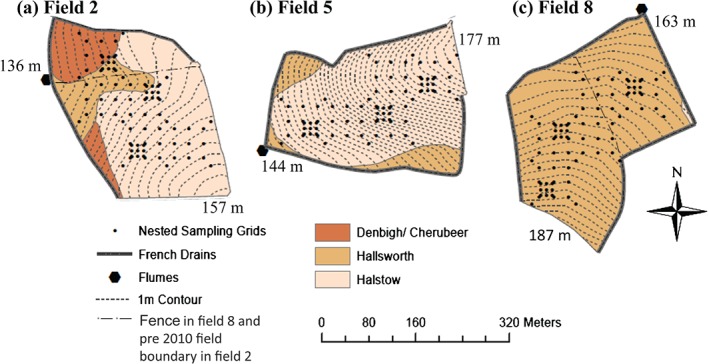
Soil sampling points, field topography, soil types (Harrod & Hogan, 2008) and French drains for: field 2 (a), field 5 (b) and field 8 (c). The pre‐2010 dividing field boundary is shown for field 2. Field 8 is divided into two parts by a fence.

The three largest fields (∼6.5–7.5 ha) in each farmlet were chosen for this study: field 2, Great Field; field 5, Orchard Dean; field 8, Higher and Middle Wyke Moor (Figure 2). Field characteristics such as soil types, topography and management are presented in Figure 2 and Table 1. Please see Peukert *et al.* (2014) for more detail.

**Table 1:** Physical site characteristics, short‐term inorganic fertilizer and farmyard manure inputs and long‐term ploughing history for the three fields sampled

Site characteristic	Field 2	Field 5	Field 8
Catchment size / ha	6.71	6.59	7.59
Mean slope (SD) / %	6.11 (1.56)	11.79 (3.1)	6.86 (1.59)
Slope range / %	0.98–9.81	5.26–20.89	3.52–13.37
French drain length / m	601.8	926.9	983.6
	Halstow (68%)		
Soil types and extent	Denbigh (18%) Hallsworth (14%)	Halstow (84.5%) Hallsworth (15.5%)	Hallsworth (99.5%) Halstow (0.5%)
Inorganic fertilizer input (2011–2012) / kg N ha^−1^ kg P ha^−1^	284 32	284 32	304 32
Farmyard manure inputs (2011–2012) / t ha^−1^	176	176	Eastern part: 0 Western part: 39.2
	Northern part: permanent grassland for ≈30 years	Permanent grassland for ≈30 years	Eastern part: last ploughed 1993
	Southern part: ploughed in 2007		Western part: permanent grassland for ≈30 years

SD, standard deviation.

The fields have been grassland for the past 30 years, but vary in their ploughing and reseeding history. Field 5, the northern part of field 2 and the eastern part of field 8 have been permanent pasture for 30 years, whereas the southern part of field 2 and the eastern part of field 8 were ploughed and reseeded in 2007 and 1993, respectively. The fields were managed conventionally for intensive cattle and sheep production, which involved grazing or grass cutting for silage (Peukert *et al.*, 2014). Inorganic fertilizer and manure inputs for each field are listed in Table 1. Inorganic fertilizer and manure were applied in accordance with the Code of Good Agricultural Practice (DEFRA, 2010) and the Nitrate Vulnerable Zone guidelines (DEFRA, 2008) and can, therefore, be considered to represent a standard management practice. Nutrient inputs were similar for each field, with minor differences that represent those typical of grassland systems.

### Soil sampling and statistical analysis

To characterize within‐ and between‐field spatial variation (objective 1), the soil was sampled in June 2012. Soil bulk density (BD), organic matter (SOM), total C (TC), total N (TN) and total P (TP) were measured.

A total of 252 soil samples were taken, 84 in each field. All measurements were made on cores from 0 to 10‐cm soil depth, the soil layer that comprises most of the soil‐plant‐water processes. Sample preparation and analysis, except for TP, followed the methods described by Peukert *et al.* (2012). Total P was determined by a sulphuric acid extraction method on finely ground samples (Saunders & Williams, 1955). In all analyses, quality control standards were used to ensure analytical quality. The ratios TC:TN, TC:TP and TN:TP were calculated for each field.

To characterize within‐field spatial variation of soil properties, a nested sampling pattern was chosen for each field. Samples were taken on a 25 m × 25 m grid, which was supplemented with 12 points at a broader scale of 75 m × 75 m and a further three sets of samples on a 10 m × 10 m grid (Figure 2).

All geostatistical analyses were undertaken with GenStat 16th edition (VSN International, Hemel Hempstead, UK). The statistical distribution of the data was determined by calculating the skewness coefficient and examining the data for outliers. We removed outliers only if they could be explained. No transformation of the data was required. Linear and quadratic surfaces were fitted to the data to identify any spatial trend present (Oliver & Webster, 2014). A trend is smooth variation where the mean changes predictably according to geographical location; it violates the assumption of randomness that underpins geostatistical theory. Experimental variograms were computed by Matheron's method of moments:

(1) $$\gamma \left(h\right)=\frac{1}{2m\left(h\right)}\sum_{j=1}^{m\left(h\right)}{\left\{z\left(x_{j}\right)-z\left(x_{j}+h\right)\right\}}^{2}\text{,}$$

where *γ*(**h**) is the semivariance, half the average of the squared differences between values, *z*(**x**_*j*_) and *z*(**x**_*j*_ + **h**) of *z* at places **x**_*j*_ and **x**_*j*_ + **h**, separated by the lag distance **h**, a vector in both distance and direction and *m*(**h**) is the number of paired comparisons at lag **h**. Different lag distances and maximum lag distances were tested to ensure that the experimental variogram was computed reliably. If the experimental variogram increases without bound and increasingly steeply at longer lag distances or has a concave form near to the origin, it might indicate a possible trend in the underlying process. Furthermore, if variograms of the residuals from the fitted linear and quadratic surfaces show spatial structure (Oliver & Webster, 2014), this would indicate a departure from stationarity and would require a different approach to compute the variogram based on residual maximum likelihood (see Webster & Oliver, 2007, for more detail).

There was no evidence of a trend in the experimental variograms of the raw data for fields 5 and 8, therefore, we fitted several variogram models to the experimental variograms, for example spherical, exponential and power functions. The models were assessed by cross‐validation and those with the smallest mean squared errors were chosen for further analyses (Oliver & Webster, 2014). The spherical model provided the best fit to all of our experimental variograms; the equation in its isotropic form is given by:

(2) $$\gamma \;\left(h\right)=c_{0}+c\;\left\{\frac{3h}{2r}-\frac{1}{2}\;{\left(\frac{h}{r}\right)}^{3}\right\},\;for\;0<h\leq r\text{,}$$

where *h* is the lag distance, *c_0_* is the nugget variance, *c* is the sill of the spatially dependent component and *r* is the range of spatial dependence. Where there was evidence of trend in the soil properties of field 2, there was little spatial structure in the residuals from the fitted surfaces.

Values between sampling sites were predicted by kriging in GenStat for eventual mapping of the spatial distribution of the variables. Values were predicted over 5 m × 5 m blocks in each field at a grid interval of 5 m. The kriged estimates were imported into arcgis 10 (ESRI, Redlands, CA, USA) for mapping. To predict soil properties with underlying trend, but no spatial structure in the residuals, inverse distance weighing was used in arcgis. To avoid over‐smoothing of the data by inverse distance interpolation, we used a maximum of four nearest neighbours only. The individual maps of prediction for each field were combined with the mosaic tool in arcgis 10 so that the values had the same colour classification.

We used boxplots to assess between‐field variation visually. It is difficult to test for differences because the samples are spatially dependent and were not sampled at random, but were sampled systematically on a grid. To identify possible underlying causes of within‐field variation: (i) known physical site characteristics (e.g. topography) and past and present management records were consulted to interpret the effects of land management on soil properties and (ii) spatial patterns were identified in the maps of prediction and compared visually with previous and current split management in each field.

### Hydrology and water quality monitoring

To characterize between‐field differences in hydrology and water quality (objective 2), monitoring was undertaken between June 2012 and April 2013. In summary, hydrology (rain and discharge) and the water quality variables TC, total oxidized nitrogen‐N (TON_N_), TP and suspended sediment (SS) were monitored by automated, semi‐automated and manual methods. Griffith *et al.* (2013) describe the instrumentation of the Farm Platform and Peukert *et al.* (2014) describe the specific instrumentation, methodology, data management and quality assurance.

Paired *t‐*tests were computed in genstat to test for between‐field differences in hydrology and water quality following Schilling *et al.* (2013). Although the data were not sampled at random, we followed others in the use of paired *t*‐tests on our time‐series measurements (Jokela & Casler, 2011; Schilling *et al*., 2013). To identify potential causal mechanisms, such as between‐field differences in soil properties, topography and resulting hydrology, the differences in soil properties and topography and hydrological characteristics were compared with the between‐field differences in nutrient losses. For example, we assessed whether the field with the largest soil TP content also had the greatest TP losses in discharge water.

To establish a suitable baseline characterization between future control and treatment sites in terms of soil and water properties (objective 3), we (i) used the results of objective 1 and (ii) determined the suitability of the sampled fields for a paired catchment approach. We correlated pollutant (TON_N_, TP and TC) concentrations in the water between pairs of fields (Schilling *et al.*, 2013) to determine whether the fields respond to rain in similar ways (comparing slopes and intercepts) in terms of flow and pollutant concentrations. The latter is an important prerequisite for paired field and catchment studies (Schilling *et al.*, 2013).

## Results

### Characterization of between‐ and within‐field soil spatial variation

Spatial variation was detected both between and within the fields sampled (objective 1). Mean soil properties were different between the fields (Table 2 and boxplots in Figure 4). Field 5 had the largest soil BD and largest SOM, TC, TN and TP concentrations. Field 8 had the smallest soil BD. Field 2 had the smallest SOM, TC and TN concentrations. Total P concentration was intermediate in field 2 and smallest in field 8. The TC:TN and TC:TP ratios were smallest in field 5, intermediate in field 2 and largest in field 8.

**Table 2:** Summary of the mean values ± standard error of the measured properties for the three fields sampled

Measured soil property	Field 2	Field 5	Field 8
Bulk density / g cm^−3^	0.89 ± 0.02	0.96 ± 0.01	0.8 ± 0.13
Total carbon / g kg^−1^	35.88 ± 0.71	49.83 ± 0.69	42.23 ± 0.67
Total nitrogen /g kg^−1^	4.25 ± 0.08	6 ± 0.07	4.81 ± 0.07
Total phosphorus / g kg^−1^	1.33 ± 0.03	1.5 ± 0.02	1.16 ± 0.06
Organic matter / g kg^−1^	88.75 ± 2.1	118.2 ± 1.34	101.1 ± 1.13
TC:TN	8.56	8.31	8.78
TC:TP	34.88	33.3	36.56
TN:TP	3.24	4.01	4.17

TC, total carbon; TN, total nitrogen; TP, total phosphorus.

**Figure 3:**
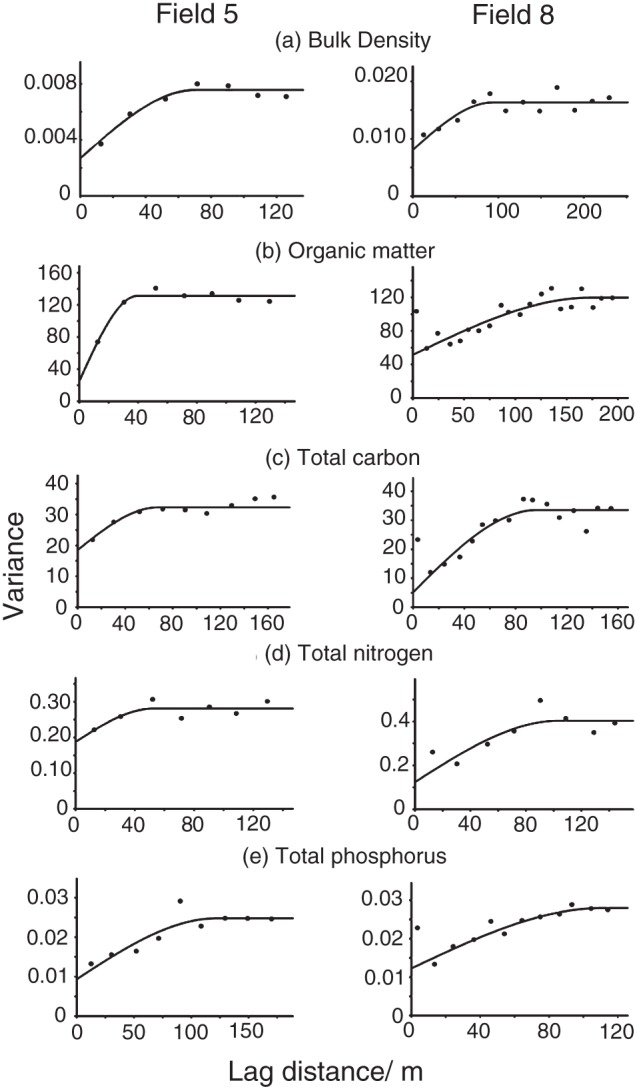
Experimental variograms and fitted models for the various soil properties for field 5 and field 8: (a) bulk density, BD, (b) soil organic matter, SOM, (c) total carbon, (d) total nitrogen and (e) total phosphorus. Symbols are experimental semivariances (estimated by Matheron's method of moments) and the solid line is the fitted spherical model.

**Figure 4:**
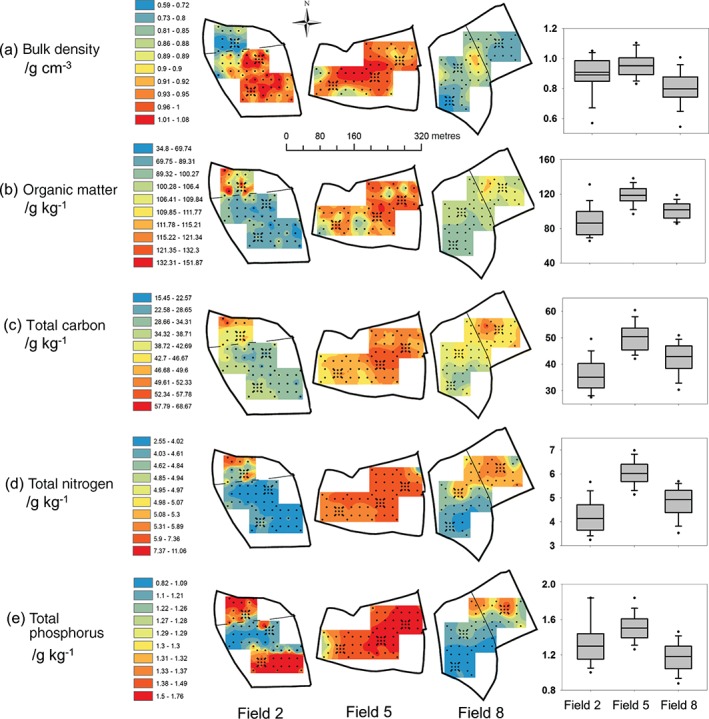
Soil spatial distribution maps made by inverse distance weighting for field 2 and by kriging for field 5 and field 8 (except for TP in field 8, which was mapped by inverse distance weighting). Boxplots show the between‐field differences in means of each soil property. The field boundary (pre 2010) in field 2 and the fence between the eastern and western parts in field 8 are shown. The boxplots indicate the lower and upper quartiles, the median and the whiskers ± 1.5 times the interquartile range. Dots indicate the 5th and 95th percentiles.

**Figure 5:**
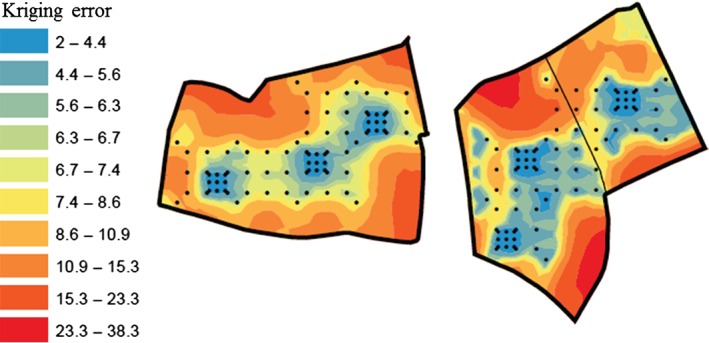
Kriging errors for total carbon for (a) field 5 and (b) field 8. The error is smallest when the sampling is most dense.

The spherical function provided the best fit to the experimental variograms of fields 5 and 8 (Figure 3 and Table 3) and these were used for kriging. Figure 4 shows the maps of the kriged predictions; these are bounded by the extent of the sampling. Kriging errors become large at the edges of the sampled area and beyond, and are smallest where the sampling is most dense (Figure 5).

**Table 3:** Parameters of fitted spherical variogram model for fields 5 and 8

Field	Variable	Nugget variance, *c*_0_	Spatially correlated sill, *c*	Range, *r*, / m	Variance explained by the model / %
5	Bulk density / g cm^−3^	0.003	0.01	72.7	64.7
5	Organic matter / g kg^−1^	27.71	104.4	41.9	36.2
5	Total carbon / g kg^−1^	17.39	14.99	67.4	42.0
5	Total nitrogen / g kg^−1^	0.18	1.20	79.4	38.2
5	Total phosphorus / g kg^−1^	0.01	0.01	129.2	58.4
8	Bulk density / g cm^−3^	0.01	0.01	93.7	59.9
8	Organic matter / g kg^−1^	51.35	68.35	173.7	79.1
8	Total carbon / g kg^−1^	5.17	28.31	96.3	83.5
8	Total nitrogen / g kg^−1^	0.12	0.28	104.0	56.0
8	Total phosphorus / g kg^−1^	0.01	0.02	111.6	89.3

Within‐field variation of soil properties, based on the variogram ranges, occurred over distances of 41.9 m (SOM) to 129.2 m (TP) in field 5, and over distances of 93.7 m (BD) to 173.7 m (SOM) in field 8 (Table 3). Figure 4 shows that the spatial variation of the soil properties in field 5 is more uniform than that for the other two fields. Bulk density, SOM and TC concentrations (Figure 3a–c) are slightly larger in the centre of field 5 and towards the northeast. Figure 4 shows that field 8 has different patterns of variation in the eastern and western parts of the field; these areas were managed differently. The eastern part has larger SOM, TC, TN and TP concentrations (Figure 4b–e). This part of the field is also at a lower elevation and there is an area of larger BD around the fence than in the rest of the field (Figure 4a).

A quadratic south–north trend was detected in field 2 for each soil property; it explains between 30.3 and 62.8% of the variation (Table 4). There was no spatial structure in the residuals after the trend surface had been fitted. Therefore, values for field 2 (Figure 4) were predicted by inverse distance weighting and are likely to be less accurate than those predicted by kriging in fields 5 and 8. The north–south trend is evident in the maps of field 2 (Figure 4). The northern and southern parts of field 2 have different patterns of variation that relate to the historical split management. Figure 4(a–d) shows that the northern part of the field contains more SOM, TC and TN and has a smaller BD than the southern part. In contrast, TP concentrations (Figure 4e) are large in the north and south with smaller concentrations between.

**Table 4:** Quadratic trend surfaces for field 2

Variable	% variance explained by fitted trend
Bulk density / g cm^−3^	30.3
Organic matter / g kg^−1^	34.1
Total carbon / g kg^−1^	62.8
Total nitrogen / g kg^−1^	50.0
Total phosphorus / g kg^−1^	36.3

There was no spatial structure in the residuals.

### Characterization of between‐field differences in hydrology and water quality in relation to between‐field differences in soil properties and other site characteristics

The three fields were different in terms of mean discharge, mean TON_N_ and TP concentrations, field 2 was different from fields 5 and 8 in terms of suspended sediment, and field 5 was different in terms of mean TC concentrations (Table 5) (objective 3). Field 5, with the largest runoff coefficients, slope and plant nutrient concentrations (Table 2), had the largest mean nutrient concentrations and loads in its discharge water (Table 5). The relations of between‐field differences in soil properties and differences in the properties of water quality were not linear for fields 2 and 8.

**Table 5:** Hydrology and water quality characteristics for the three fields

Variable		Field 2		Field 5		Field 8	
Hydrology	Mean discharge / l s^−1^ ± SE	1.21 ± 0.0001	*N* = 35 712	1.46 ± 0.0002	*N* = 35 712	1.55 ± 0.0002	*N* = 35 712
	% of rain as discharge year^−1^	40.47		53.9		46.22	
Suspended sediment	Annual yield / kg ha^−1^ year^−1^	182.2–194.3		433.9–527.4		213.4–220.9	
	Mean concentration / mg l^−1^ ± SE	13.36 ± 0.0018	*N* = 11 546	14.89 ± 0.003	*N* = 10 413	15.1 ± 0.0016	*N* = 11 435
	Mean load / mg s^−1^ ± SE	103.5 ± 0.04		159.0 ± 0.09		119.5 ± 0.05	
Total oxidized nitrogen‐N	Annual yield / kg ha^−1^ year^−1^	1.4–1.8		2.9–3		0.9–1	
	Mean concentration / mg l^−1^ ± SE	0.88 ± 0.0001	*N* = 12 972	1.17 ± 0.0001	*N* = 14 258	0.45 ± 0.0003	*N* = 12 168
	Mean load / mg s^−1^ ± SE	0.64 ± 0.0001		1.006 ± 0.0001		0.40 ± 0.0001	
Total phosphorus	Annual yield / kg ha^−1^ year^−1^	0.42		0.87		0.44	
	Mean / µg l^−1^ ± SE	48.86 ± 0.006	*N* = 4405	55.28 ± 0.023	*N* = 2221	47.57 ± 0.018	*N* = 2383
	Mean load / µg s^−1^ ± SE	202.8 ± 0.14		607.25 ± 0.76		447.7 ± 0.49	
Total carbon	Annual yield / kg ha^−1^ year^−1^	122.2		179.1		109.4	
	Mean concentration / mg l^−1^ ± SE	23.65 ± 0.05	*N* = 173	25.76 ± 0.005	*N* = 181	17.84 ± 0.029	*N* = 246
	Mean load / mg s^−1^ ± SE	359.8 ± 1.86		476.53 ± 2.7		351.3 ± 1.314	

Mean values for each variable monitored are shown for each field. For water quality variables, mean concentrations, loads and annual yields are also shown to illustrate differences between the responses of each field to rainfall. Significant differences are discussed in the Results section.

Hydrology and water quality monitoring was carried out from April 2012 to March 2013, with sampling resolutions up to every 15 minutes. N, the number of measurements available to calculate means; SE, standard error.

### Establishment of a suitable baseline characterization between future control and treatment sites

The results of objective 1 characterize the soil baseline and fields were paired to establish a baseline in terms of hydrology and water quality (objective 3). The fields were different in terms of soil characteristics (Table 2). The paired future control and treatment fields were strongly correlated in terms of rain (*r* ≈ 0.84), which suggests that rain occurs at the same time because of close proximity, and discharge (*r* ≈ 0.95), which indicates that the fields' hydrology is similarly affected by rain (Table 6). The paired future control and treatment fields had strong correlations for the water quality properties (*r* = 0.68–0.83) (Table 6), which suggests that mobilization and delivery of SS, TON_N_, TP and TC occur at the same time throughout the time series.

**Table 6:** Correlation coefficients (r) for each water quality variable between the selected control field (field 5) and the two future treatment fields (field 2 and field 8)

Variable	Fields 5 and 2	Fields 5 and 8
Precipitation	*r* = 0.9, *N* = 35 713	*r* = 0.9, *N* = 35 713
Discharge	*r* = 0.96, *N* = 35 713	*r* = 0.94, *N* = 35 713
Suspended sediment	*r* = 0.77, *N* = 8 178	*r* = 0.83, *N* = 7 964
Total oxidized nitrogen‐N	*r* = 0.79, *N* = 11 045	*r* = 0.68, *N* = 10 988
Total phosphorus	*r* = 0.75, *N* = 1 886	*r* = 0.71, *N* = 2 026
Total carbon	*r* = 0.82, *N* = 59	*r* = 0.7, *N* = 85

N, the number of measurements available for each correlation.

## Discussion

### Characterization of between‐field and within‐field spatial variation

The fields were different in terms of soil physical properties and nutrient concentrations, in spite of similar management for at least 6 years prior to the study, and they also showed within‐field soil spatial variation (objective 1).

The soil in the field with the longest time since last ploughing (field 5, at least 30 years) contained the largest concentrations of SOM, TC and TN. The field with an intermediate time since last ploughing (field 8, western part not ploughed for at least 30 years, eastern part ploughed in 1993) had intermediate SOM, TC and TN contents. The field that had been ploughed most recently (field 2, ploughed in 2007) had the smallest concentrations of SOM, TC and TN. Ploughing reduces SOM, TC and TN in soil by breaking down soil aggregates, mixing the topsoil with deeper soil layers and thereby increasing soil aeration, which exposes previously protected SOM to mineralization and increases decomposition of SOM and microbial turnover (Liu *et al*., 2006). In contrast, nutrients accumulate at the surface of permanent grasslands because they are applied to the soil surface and not mixed with deeper soil layers (Schärer *et al*., 2007).

These results suggest that agricultural fields with different management histories (more than 6 years), but similar recent land use, cannot be assumed to be similar in their mean soil properties. Therefore, long‐term management history should be included when interpreting soil data at this scale.

The variogram parameters of fields 5 and 8 (Table 3) suggest different within‐field patterns of soil spatial variation and also differences between soil properties (Figure 4). The ranges of spatial dependence over which variation occurs on the Farm Platform, compare with those reported for a single field in an earlier publication (Peukert *et al.*, 2012) and those reported for other intensively managed temperate grasslands (McCormick *et al.*, 2009). Such within‐field patterns might be affected by different management histories, but also by differences in topography that affect the movement of water and soil downslope. For example, the marked north–south trend in all soil properties in field 2 might reflect management effects such as different ploughing management of the two field areas, topography and resultant water movement downslope towards the south with the likely re‐deposition of soil and nutrients. One or a combination of the following mechanisms might be responsible for the differences between the two parts of field 8 that are divided by a fence. The western part has the more recent ploughing history (last ploughed in 1993), whereas the eastern part has not been ploughed for approximately 30 years, which might have resulted in the accumulation of SOM, TC, TN and TP. Different short‐term nutrient management strategies (FYM applications in the eastern part, but not in the western part, 1 year prior to sampling) might also have increased soil SOM, TC, TN and TP concentrations (Dungait *et al.*, 2012). In addition, the direction of water movement through the field and consequent soil erosion down the slope, in general from the western to the eastern part, is likely to have re‐deposited SOM, TC, TN and TP in the east. In contrast, the central eastern area in field 5 with large concentrations of SOM, TC, TN and TP and a large BD coincided with the area where cattle congregate. The elevated BD may be caused by livestock trampling and the large concentrations of nutrients and SOM by manure inputs from the grazing livestock (Page *et al.*, 2005; Bilotta *et al*., 2007).

### Characterization of between‐field differences in hydrology and water quality in relation to between‐field differences in soil properties and other site characteristics

Table 5 shows that the three fields have marked differences in terms of their nutrient concentrations (objective 1) and their mobilization potential (objective 2), which is given by the slope and runoff coefficient. The latter reflects differences in nutrient and sediment losses between the fields. Long‐term management differences have affected soil properties and changed soil processes such as nutrient cycling within and between the fields.

A long history of permanent grassland is generally considered to be the ‘best’ agricultural management option in terms of diffuse pollution. However, the field that has the longest history of permanent grassland in this research had the greatest sediment and nutrient losses, most of which exceeded the amounts of sediment and nutrients allowed under EU and UK water quality standards (Peukert *et al*., 2014). This might be explained by a combination of (i) the largest nutrient concentrations are associated with the surface accumulation of nutrients applied over time and available for mobilization and (ii) the largest mobilization potential of those nutrients to be moved. The faster the rainwater moves over or through the soil, the greater is the force of that water. Therefore, the greater is the potential of the water to erode soil and move TP and TC attached to soil particles or large organic matter particles (Gächter *et al*., 2004; Bilotta *et al*., 2010 Glendell & Brazier, 2014; Peukert *et al*., 2014). Field 5 had the largest dissolved mineral N pool, indicated by the smallest soil TC:TN ratio, which might explain why the field had the largest TON_N_ loss. The smaller the soil TC:TN ratio, the greater is the turnover of SOM, which increases the available nitrate pool in the soil (Mooshammer *et al*., 2012). The fact that the field with the longest history of permanent pasture is the most polluting should be considered in land management guidelines and advice for future compliance with surface water quality standards. Permanent grassland management alone cannot be considered a means to resolve erosion and runoff issues in agricultural land. More detailed advice is needed, for example on how to reduce nutrient inputs and how to alleviate compaction in grasslands by aeration.

Long‐term management effects that go back at least 6 years, but possibly up to 30 years ago or longer, were still evident in soil properties and resultant water quality. Such long response times are known to occur (Burt *et al.*, 2011). Full SOM turnover times have been estimated to take approximately 36 years (Balesdent *et al.*, 1987), and long‐term surface nutrient accumulations in grasslands can have a long‐lasting effect on elevated nutrient concentrations in surface waters (Schärer *et al.*, 2007). Therefore, there might be decadal time lags between agricultural management and its effects on soil and water quality. Such long‐term effects of management might indicate how long it can take for water quality to improve after the implementation of mitigation measures (decades). Measures implemented over the past few years might still be effective in the future. Consequently, our expectations on the time it takes for water quality to improve need to be adjusted where long‐term monitoring is required.

Differences in nutrient status of the soil between the fields, the spatial variation of properties within the fields and differences in resulting water quality between fields confirm that intensively managed grassland fields, even on similar soil types, cannot be treated as homogeneous management units. Water quality, productivity and economic benefits might result from sampling the soil of every field and matching nutrient inputs to their status in the soil. Furthermore, it might be beneficial to take into account the existing spatial variation within fields, which is done with precision farming in arable areas. For example, the two parts of fields 2 and 8 could be treated as different management units (McCormick *et al.*, 2009). The variogram ranges from our research suggest sampling intervals of 13–60 m (approximately a third of the range of spatial dependence as a ‘rule of thumb’), depending on the soil property of interest. Sampling at resolutions of < 50 m would be time‐consuming and costly, but the commonly used ‘W‐pattern’ across a field to obtain a single bulked sample will not reveal any spatial variation. To optimize sampling, farmers could take a systematic approach to sampling based on the results in this paper. Areas with different management histories, different soil types or differences in crop yield could be sampled strategically.

### Establishment of a suitable calibration between future control and treatment sites

The monitoring of soil and water quality provides a robust baseline for future comparisons and for future paired catchment studies.

This research has shown that the fields are suitable for a paired catchment approach, which does not require fields to have the same nutrient concentration in soil or discharge water. The fields must, however, respond to rain similarly in terms of the generation of discharge and the behaviour of pollutants with discharge (tested by correlation of time series) (Jokela & Casler, 2011; Schilling *et al*., 2013). The correlations between the future control field (5) and future treatment fields (2 and 8) were strong in terms of hydrology and water quality properties throughout the entire time series (Schilling *et al.*, 2013).

The baseline characterization presented here will enable a robust comparison of future soil and water quality with their baseline qualities. Any change in mean soil properties and their spatial variation and distribution within fields, together with any change in the components of the soil to the water source‐mobilization‐delivery continuum, can be considered as future management effects with some confidence (Jokela & Casler, 2011; Wall *et al.*, 2011; Schilling *et al.*, 2013).

## Conclusions

The results from this research have the following important implications.

- Agricultural fields with different management histories, but the same land use, cannot be assumed to be similar in terms of soil properties or their spatial distribution, which emphasizes the importance of baseline characterization and paired catchment studies. Past management can have a long‐term effect on soil properties and diffuse losses. Long‐term management must be taken into account when soil and water quality data are interpreted.
- The baseline characterization will enable the identification of future changes in management with some confidence.
- Permanent grasslands require careful management to avoid accumulation of surface nutrients and soil compaction. Benefits in potential herbage yield and water quality could be gained from intensive soil sampling of every field to enable site‐specific management.
- Permanent grasslands will not necessarily reduce diffuse pollution. Long‐term effects of management indicate that it can take decades for water quality to improve.

## References

[ejss12351-bib-0001] Balesdent, J. , Mariotti, A. & Guillet, B. 1987 Natural ^13^C abundance as a tracer for studies of soil organic matter dynamics. Soil Biology & Biochemistry, 19, 25–30.

[ejss12351-bib-0002] Bilotta, G.S. , Brazier, R.E. & Haygarth, P.M. 2007 The impacts of grazing animals on the quality of soils, vegetation, and surface waters in intensively managed grasslands. Advances in Agronomy, 94, 237–280.

[ejss12351-bib-0003] Bilotta, G.S. , Krueger, T. , Brazier, R.E. , Butler, P. , Freer, J. , Hawkins, J.M.B. *et al.* 2010 Assessing catchment‐scale erosion and yields of suspended solids from improved temperate grassland. Journal of Environmental Monitoring, 12, 731–739. 20445863 10.1039/b921584k

[ejss12351-bib-0004] Breuer, L. , Huisman, J.A. , Keller, T. & Frede, H.‐G. 2006 Impact of a conversion from cropland to grassland on C and N storage and related soil properties: analysis of a 60‐year chronosequence. Geoderma, 133, 6–18.

[ejss12351-bib-0005] Burt, T.P. , Howden, N.J.K. , Worall, F. , Whelan, M.J. & Bieroza, M. 2011 Nitrate in United Kingdom rivers: policy and its outcomes since 1970. Environmental Science & Technology, 45, 175–181. 20681541 10.1021/es101395s

[ejss12351-bib-0006] DEFRA 2008 Guidance on Complying with the Rules for Nitrate Vulnerable Zones in England for 2013–2016 [WWW document]. URL https://www.gov.uk/government/uploads/system/uploads/attachment_data/file/432141/pb14050‐nvz‐guidance.pdf [accessed on 20 March 2016].

[ejss12351-bib-0007] DEFRA 2010 Fertiliser Manual (RB209) [WWW document]. URL http://www.ahdb.org.uk/documents/rb209‐fertiliser‐manual‐110412.pdf [accessed on 20 March 2016].

[ejss12351-bib-0008] Dungait, J.A.J. , Cardenas, L.M. , Blackwell, M.S. , Wu, L. , Withers, P.J.A. , Chadwick, D.R. *et al.* 2012 Advances in the understanding of nutrient dynamics and management in UK agriculture. Science of the Total Environment, 434, 39–50. 22748430 10.1016/j.scitotenv.2012.04.029

[ejss12351-bib-0009] Gächter, R. , Steingruber, S.M. , Reinhardt, M. & Wehrli, B. 2004 Nutrient transfers from soil to surface waters: differences between nitrate and phosphate. Aquatic Sciences, 66, 117–122.

[ejss12351-bib-0010] Glendell, M. & Brazier, R.E. 2014 Accelerated export of sediment and carbon from a landscape under intensive agriculture. Science of the Total Environment, 476‐477, 643–656. 10.1016/j.scitotenv.2014.01.057 24503335

[ejss12351-bib-0011] Glendell, M. , Granger, S.J. , Bol, R. & Brazier, R.E. 2014 Quantifying the spatial variability of soil physical and chemical properties in relation to mitigation of diffuse water pollution. Geoderma, 214, 25–41.

[ejss12351-bib-0012] Griffith, B. , Hawkins, J.M.B. , Orr, R.J. , Blackwell, M.S.A & Murray, P.J . 2013 The North Wyke farm platform: methodologies used in remote sensing of the water quantity and quality of drainage water In: Water Resources and Catchment Management in Grassland and Forage Systems (eds Michalk D.L., Millar G.D., Badgery W.B. & K.M. Broadfoot), pp. 1453–1455. New South Wales Department of Primary Industry, Sydney.

[ejss12351-bib-0013] van Grinsven, H.J.M. , ten Berge, H.F.M. , Dalgaard, T. , Fraters, B. , Durand, P. , Hart, A. *et al.* 2012 Management, regulation and environmental impacts of nitrogen fertilization in Northwestern Europe under the Nitrates Directive; a benchmark study. Biogeosciences Discussions, 9, 7353–7404.

[ejss12351-bib-0014] Harrod, T.R. & Hogan, D.V. 2008 The Soils of North Wyke and Rowden [WWW document]. URL http://www.rothamsted.ac.uk/sites/default/files/SoilsNWRowden.pdf [accessed on 20 March 2016].

[ejss12351-bib-0015] Horrocks, C.A. , Dungait, J.A.J. , Cardenas, L.M. & Heal, K.V. 2014 Does extensification lead to enhanced provision of ecosystem services from soils in UK agriculture. Land Use Policy, 38, 123–128.

[ejss12351-bib-0016] Johnson, D. , Hodgkinson, R. , Lord, E. , Silgram, M. , Cottrill, B. , Gooday, R. *et al* 2011 Nitrates Directive Consultation Document, The Evidence Base for Assessing the Impacts of the NVZ Action Programme on Water Quality across England and Wales [WWW document]. URL https://www.gov.uk/government/uploads/system/uploads/attachment_data/file/82408/20111220nitrates‐directive‐consult‐evid1.pdf [accessed on 19 February 2016].

[ejss12351-bib-0017] Jokela, W. & Casler, M. 2011 Transport of phosphorus and nitrogen in surface runoff in a corn silage system: paired watershed methodology and calibration period results. Canadian Journal of Soil Science, 91, 479–491.

[ejss12351-bib-0018] Liu, X. , Herbert, S.J. , Hashemi, A.M. , Zhang, X. & Ding, G. 2006 Effects of agricultural management on soil organic matter and carbon transformation‐ a review. Plant, Soil and Environment, 52, 531–543.

[ejss12351-bib-0019] McCormick, S. , Jordan, C. & Bailey, J.S. 2009 Within and between‐field spatial variation in soil phosphorus in permanent grassland. Precision Agriculture, 10, 262–276.

[ejss12351-bib-0020] Mooshammer, M. , Schnecker, J. , Wild, B. , Leitner, S. , Hofhansl, F. , Blochl, A. *et al.* 2012 Stoichiometric control of nitrogen and phosphorus cycling in decomposing beech leaf litter. Ecology, 93, 770–782. 22690628 10.1890/11-0721.1

[ejss12351-bib-0021] Oliver, M.A. & Webster, R. 2014 A tutorial guide to geostatistics: computing and modelling variograms and kriging. Catena, 113, 56–69.

[ejss12351-bib-0022] Page, T. , Haygarth, P.M. , Beven, K.J. , Joynes, A. , Butler, T. , Keeler, C. *et al.* 2005 Spatial variability of soil phosphorus in relation to the topographic index and critical source areas: sampling for assessing risk to water quality. Journal of Environmental Quality, 34, 2263–2277. 16275728 10.2134/jeq2004.0398

[ejss12351-bib-0023] Peukert, S. , Bol, R. , Roberts, W. , Macleod, C.J.A. , Murray, P.J. , Dixon, E.R. *et al.* 2012 Understanding spatial variability of soil properties: a key step in establishing field‐ to farm‐scale agro‐ecosystem experiments. Rapid Communications in Mass Spectrometry, 26, 2413–2421. 22976208 10.1002/rcm.6336

[ejss12351-bib-0024] Peukert, S. , Griffith, B.A. , Murray, P.J. , Macleod, C.J.A. & Brazier, R.E. 2014 Intensive management in grasslands causes diffuse water pollution at the farm‐scale. Journal of Environmental Quality, 43, 2009–2023. 25602218 10.2134/jeq2014.04.0193

[ejss12351-bib-0025] Saunders, W.M.H. & Williams, E.G. 1955 Observations on the determination of total organic phosphorus in soils. Journal of Soil Science, 6, 254–267.

[ejss12351-bib-0026] Schärer, M. , Stamm, C. , Vollmer, T. , Frossard, E. , Oberson, A. , Flühler, H. *et al.* 2007 Reducing phosphorus losses from over‐fertilized grassland soils proves difficult in the short term. Soil Use & Management, 23, 154–164.

[ejss12351-bib-0027] Schilling, K.E. , Jones, C. & Seeman, A. 2013 How paired is paired? Comparing nitrate concentrations in three Iowa drainage districts. Journal of Environmental Quality, 42, 1412–1421. 24216419 10.2134/jeq2013.03.0085

[ejss12351-bib-0028] Wall, D. , Hordan, P. , Melland, A.R. , Mellander, P.‐E. , Buckley, C. , Reaney, S.M. *et al.* 2011 Using the nutrient transfer continuum concept to evaluate the European Union Nitrates Directive National Action Programme. Environmental Science & Policy, 14, 664–674.

[ejss12351-bib-0029] Webster, R . & Oliver, M.A . 2007 Geostatistics for Environmental Scientists, 2nd edn. Wiley, Chichester.

